# Response to tyrosine kinase inhibitors in lung adenocarcinoma with the rare epidermal growth factor receptor mutation S768I and G724S: A case report and literature review

**DOI:** 10.1111/1759-7714.13606

**Published:** 2020-08-09

**Authors:** Cuicui Zhang, Li Lin, Ran Zuo, Yajie Wang, Peng Chen

**Affiliations:** ^1^ National Clinical Research Center for Cancer Tianjin Medical University Cancer Institute & Hospital Tianjin Japan; ^2^ Key Laboratory of Cancer Prevention and Therapy Tianjin Medical University Cancer Institute & Hospital Tianjin Japan; ^3^ Tianjin Clinical Research Center for Cancer Tianjin Medical University Cancer Institute & Hospital Tianjin Japan; ^4^ Department of Thoracic Oncology Tianjin Medical University Cancer Institute & Hospital Tianjin Japan

**Keywords:** EGFR G724S, EGFR S768I, epidermal growth factor receptor (EGFR), rare mutation

## Abstract

Mutations in the epidermal growth factor receptor (EGFR) are drivers of a subset of lung cancers. In recent years, the treatment of non‐small cell lung cancer (NSCLC), especially with EGFR inhibitors, has made rapid progress, and the median progression‐free survival (PFS) of patients with *EGFR* gene‐sensitive mutations has been significantly prolonged. However, the response effect of some uncommon *EGFR* mutations to tyrosine kinase inhibitors (TKIs) remains unclear.

Here, we present a patient with multiple *EGFR* mutations that highlights tumor heterogeneity leading to a mixed molecular response to targeted drugs and emphasizes the complexity of EGFR‐driven lung cancer. He received chemotherapy and molecular‐targeted treatment including icotinib, afatinib, osimertinib and afatinib + osimertinib.

In conclusion, patients with lung adenocarcinoma harboring the *EGFR* S768I and G724S mutations appear less sensitive to icotinib than patients with sensitive EGFR. However, the patient in our report benefited from treatment with afatinib. Here, we hope to provide information for the treatment of rare and compound mutations in patients.

## Graphical abstract

Patients with lung adenocarcinoma harboring the EGFR S768I and G724S mutations appear less sensitive to first‐generation tyrosine kinase inhibitors than patients with sensitive EGFR.However, the patient benefited from treatment with afatinib.

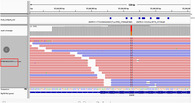

## Introduction

At present, the incidence and mortality of lung cancer have become the leading cause of cancer‐related deaths worldwide.^1^ The incidence of lung cancer in China has reached 61.4/100000. Lung cancer can be divided approximately into non‐small cell lung cancer (NSCLC) and small cell lung cancer (SCLC) according to the pathological type. NSCLC accounts for approximately 85% of lung cancer cases.^2^ Adenocarcinoma is the most common histological type of NSCLC and can be subdivided into different clinically relevant molecular subtypes according to its driver gene mutation type. One of the common mutations is epidermal growth factor receptor (EGFR) mutation.^3^ This mutation is more prevalent in female patients who do not smoke. EGFR is a well‐characterized driver of a subset of lung cancers, with activating alterations predicting sensitivity to epidermal growth factor receptor tyrosine kinase inhibitors (EGFR‐TKIs) reported in 10%–35% of lung adenocarcinomas.^4^, ^5^ EGFR plays an important role in the proliferation, growth, repair and survival of tumor cells. Mutations in the EGFR tyrosine kinase region mainly occur in exons 18–21. *EGFR* mutations are mainly concentrated in exon 19 deletion and exon 21 L858R point mutation, which are the largest beneficiaries of EGFR‐TKI molecular targeted therapy and have been unanimously verified in various clinical studies. In recent years, the prognosis of NSCLC has been improved substantially by the use of EGFR‐TKIs. These include first‐generation EGFR‐TKIs (gefitinib, erlotinib and icotinib), second‐generation EGFR‐TKIs (afatinib), and third‐generation EGFR‐TKIs (osimertinib). Currently, the first‐generation tyrosine kinase inhibitors (TKIs) gefitinib, icotinib, erlotinib, and second‐generation afatinib are commonly recommended as first‐line systemic therapies for patients harboring sensitizing *EGFR* mutations; osimertinib has also been approved for the first‐line treatment of patients with *EGFR* mutations. However, the efficacy of targeted therapy in NSCLC patients with rare *EGFR* mutations has not been verified by large‐scale clinical studies. There is no consensus on the choice of molecular‐targeted drugs. For instance, the rare exon 20 mutation S768I occurs in 1%–2% of *EGFR* mutation lung cancers.^6^ Its sensitivity to EGFR‐TKIs has been controversial; in vitro studies reported relative resistance.^7^ A secondary EGFR‐T790M mutation in exon 20 of the *EGFR* gene is frequently induced by drug selection, leading to resistance towards EGFR inhibitors. Approximately 50% of patients developed this mutation after resistance to first‐generation EGFR‐TKIs. Osimertinib is a mutant‐selective TKI approved for the treatment of T790M‐positive NSCLC and has significantly greater efficacy than platinum therapy plus pemetrexed in patients with T790M‐positive advanced NSCLC.^8^ A case report by Oztan *et al*. reported the emergence of the *EGFR* G724S mutation in *EGFR* mutant lung adenocarcinoma post progression on osimertinib.^9^ Interestingly, this G724S mutation was detected in an NSCLC patient just at the time of diagnosis, along with the exon 20 mutation S768I. It is that rare of an event. Here, we present the case of a patient with multiple *EGFR* mutations that highlights tumor heterogeneity leading to a mixed response to molecular‐targeted drugs and emphasizes the complexity of EGFR‐driven lung cancer. In this study we hope to provide information for the treatment of rare and compound mutations in patients.

## Case report

**Figure 1:**
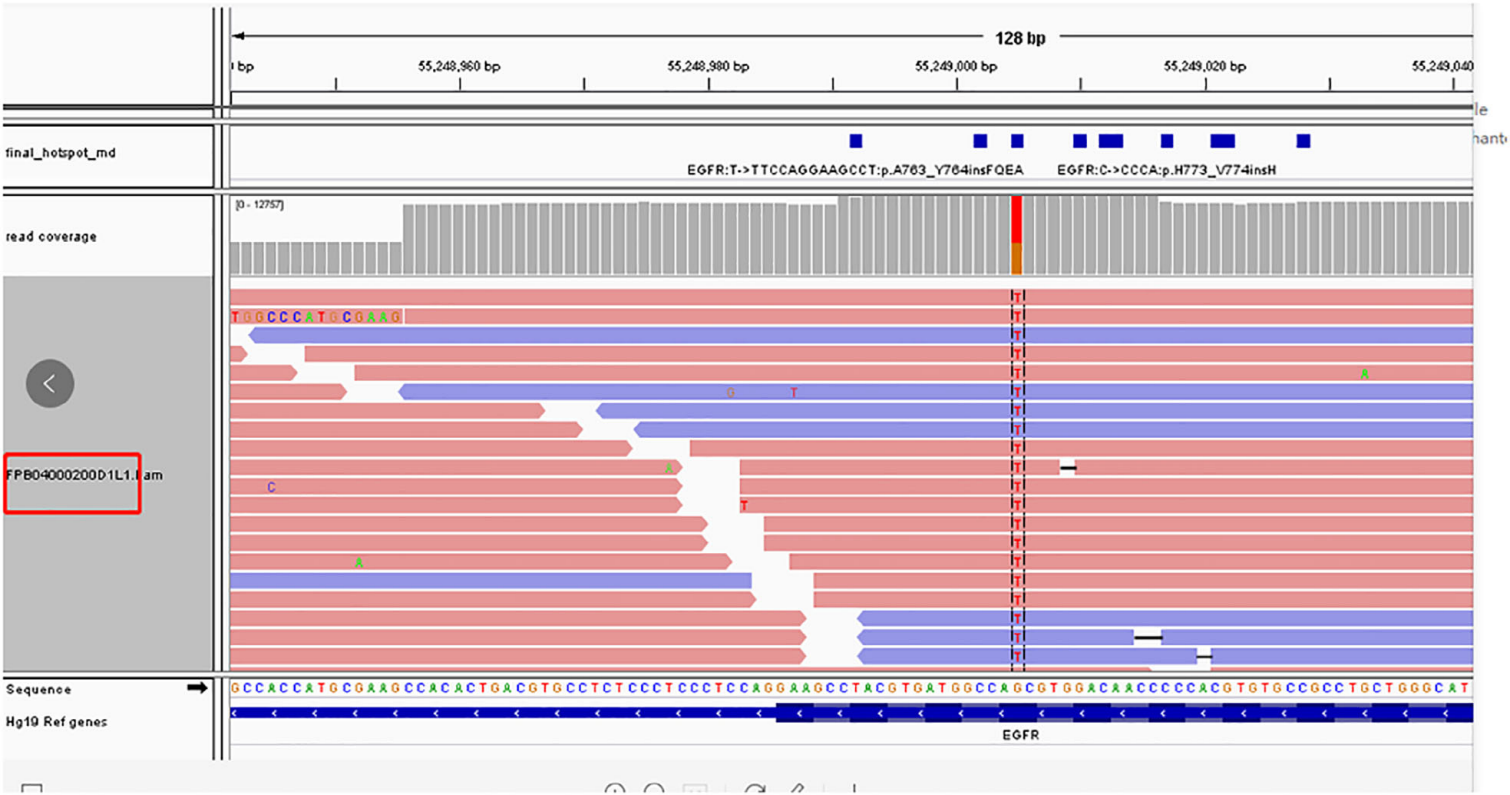
The results from gene detection.

**Figure 2:**
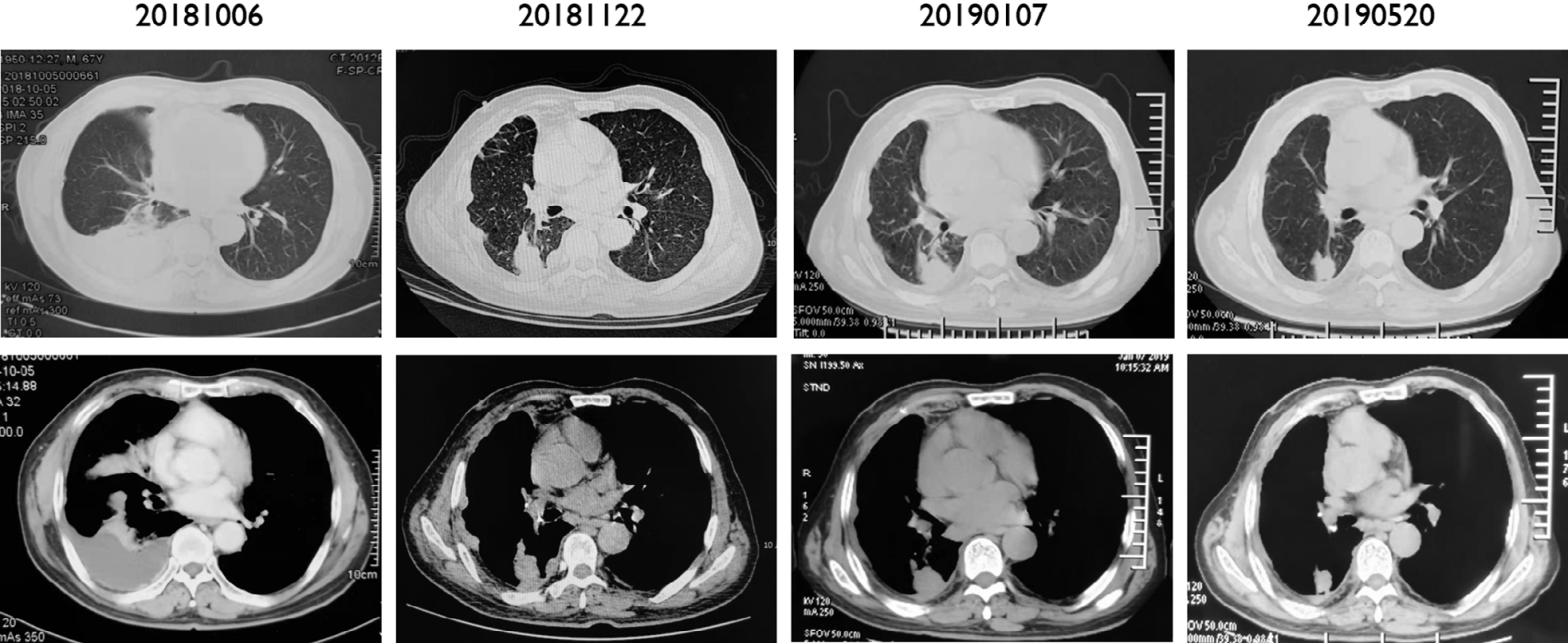
The CT scan during the treatment. “20181006” were the imaging findings before chemotherapy; “20181122” were the imaging findings after chemotherapy (before icotinib); “20190107” were the imaging findings after icotinib (before afatinib); “20190520” were the imaging findings 3 months after afatinib.

**Figure 3:**
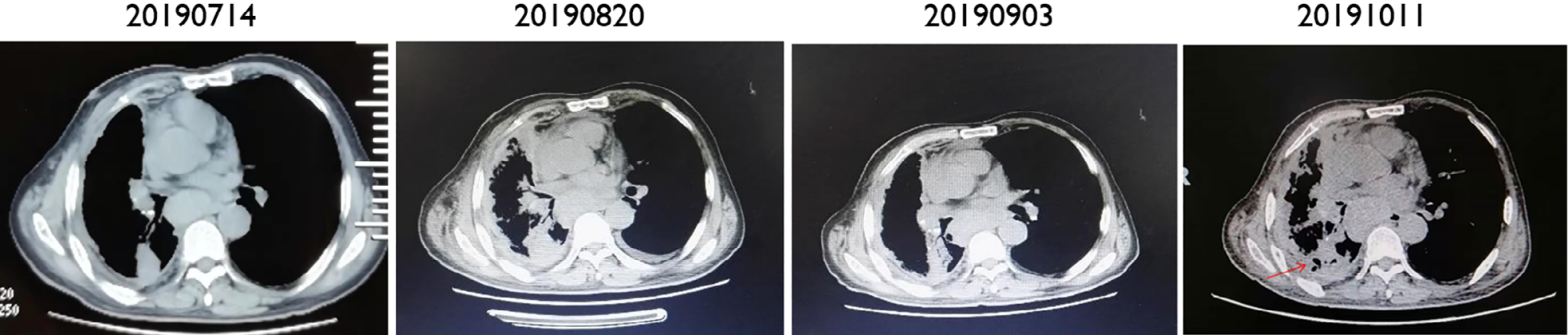
The CT scan during the treatment. “20190714” were the imaging findings 6 months after afatinib (before osimertinib); “20190820” were the imaging findings 1 month after osimertinib (before osimertinib + afatinib); “20190903” and “20191011” were the imaging findings 1 month and 2 months after osimertinib + afatinib.

**Figure 4:**
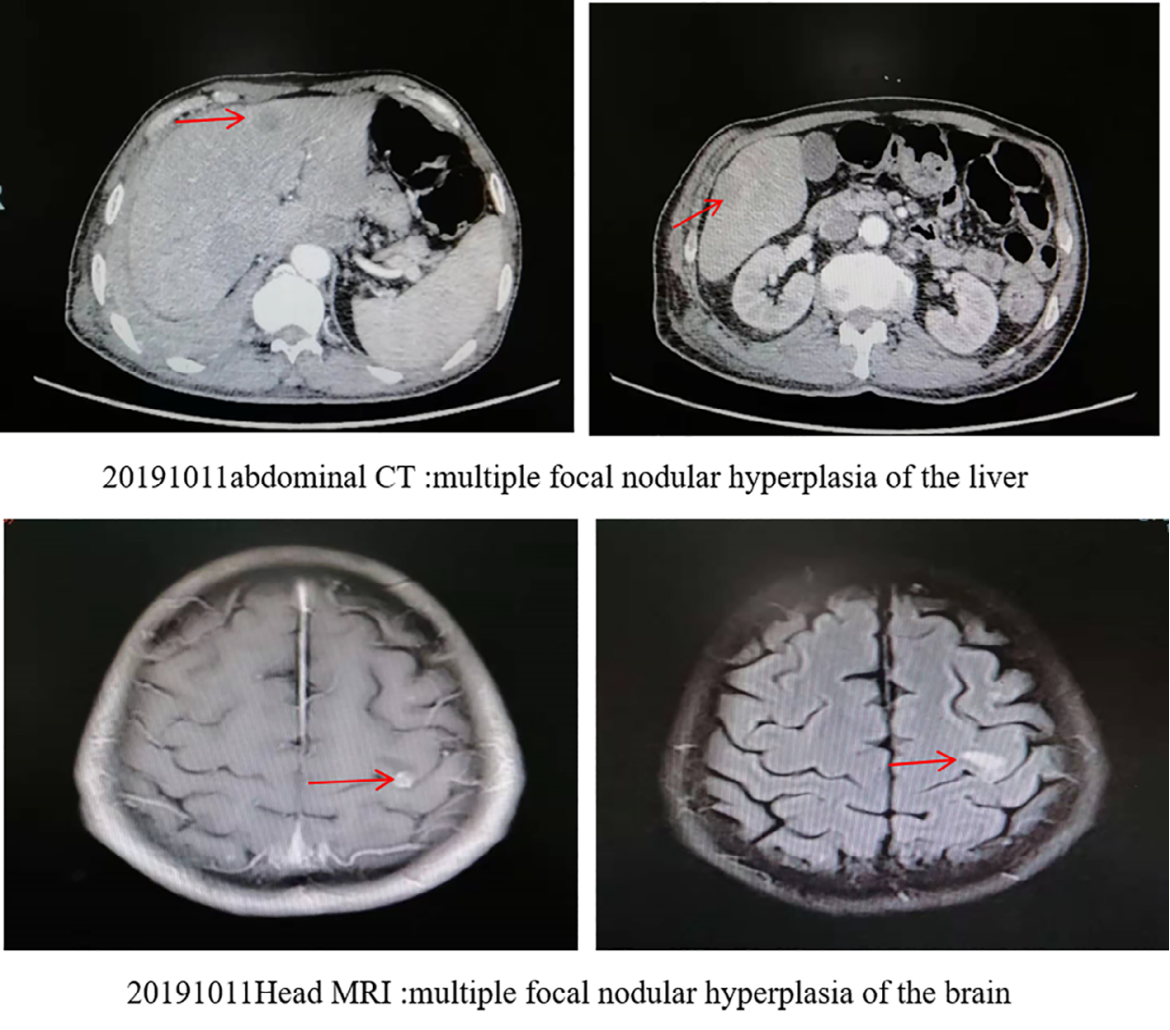
The CT scan findings two months after treatment with osimertinib and afatinib. The red arrow indicates liver and brain metastases.

A 73‐year‐old male with a 50‐year history of smoking 10 cigarettes per day, and a previous history of coronary artery disease, hypertension, and resection of rectal carcinoma, presented with a cough with phlegm and tightness in his chest. There was no family history of carcinoma. Physical examination revealed palpable bilateral enlarged lymph nodes in his neck, the largest was approximately 1 cm, firm, nontender, with unclear boundaries; and right lung sounds were slightly quieter than normal. CT images revealed a tumor in the right lobe with a malignant pleural effusion, extensive mediastinal and hilar lymphadenopathy, contralateral pulmonary and pleural metastases. The patient was diagnosed with stage IV lung adenocarcinoma. High‐throughput sequencing of the initial pleural fluid specimen revealed *EGFR* exon 18 G724S (57.5%) and exon 20 S768I (60.5%) mutations (Fig 1). The patient was treated with three cycles of carboplatin and pemetrexed as the first‐line treatment. He achieved partial response (PR) after two cycles of chemotherapy, and his cough, phlegm and chest tightness were significantly relieved. After a third cycle of chemotherapy (two months later), the patient developed weak loss of appetite. It was suggested that he should receive pemetrexed alone, but the patient believed he was intolerant to chemotherapy and refused treatment. The patient was then given treatment with icotinib (125 mg, taken orally, three times a day) after three cycles of chemotherapy, and there were no intolerable adverse reactions. Unfortunately, a CT scan showed a trend of progressive disease (PD) after a month, and the patient was switched to afatinib (40 mg, taken orally, daily). He suffered adverse reactions to this treatment which included moderate diarrhea, mild rash and mild paronychia. However, he had achieved a partial response (PR) three months after treatment initiation. The drug dosage was reduced to 30 mg, taken orally, daily because of the intolerance of adverse reactions. A CT scan performed approximately six months after initiation of afatinib revealed an increase in size of the right lower lobe nodules indicative of progressive disease (PD) and he had also developed brain metastases. We then undertook high‐throughput gene testing of peripheral blood. The results showed *EGFR* exon 18 G724S (20.67%), exon 20 S768I (20.11%), and 790M (5.01%) mutations. Osimertinib was given initially (80 mg daily). However, the patient appeared to suffer with heavy coughing after 15 days of treatment, and a CT scan showed a trend of disease progression. The patient was then switched to osimertinib (80 mg daily) combined with afatinib (30 mg daily), and surprisingly achieved complete remission of his cough after 15 days, and CT examination suggested a stable condition. However, re‐examination indicated progression of the disease and he was found to have developed metastases in the liver (see Figs 2, 3, 4 for the CT scan imaging findings). The patient was then treated with anlotinib (12 mg, orally once daily for 14 days every three weeks). At the same time CT scan examination and tumor markers were checked at intervals, and the results indicated that the lesions were in a stable condition, PFS was eight months but as a result of the significant disruption being caused by the COVID‐19 pandemic the patient was checked regularly at a local hospital, so there was no available follow‐up imaging data.

## Discussion

Lung cancer is the leading cause of cancer‐related mortality worldwide, accounting for 20% of all cancer‐related deaths.^10^, ^11^ *EGFR* mutation frequency in patients with NSCLC is relatively high (Asia, 47%; North America, 22%; and Europe, 15%). ^11^ Most of the patients are female, non‐ or mild smokers. The structure of the *EGFR* gene is mainly divided into the extracellular ligand binding domain, transmembrane regions and intracellular tyrosine kinase domain. It is composed of 28 exons. The most frequent *EGFR* mutations found by traditional or comprehensive molecular profiling of lung adenocarcinomas include indels of exon 19 (the exon 19 deletion delE746_A750 being the most common, followed by delE746_S752insV) and the exon 21 L858R point mutation, accounting for 90% of mutations. ^12^ In addition to the two most common mutations, other mutations have been found in the region between exons 18–21 of the *EGFR* gene. The current study showed that prevalence of *EGFR* S768I mutation in Chinese patients with lung AC was 0.52%. Different types of mutations have different effects on the clinical efficacy of the EGFR tyrosine kinase coding region. Many studies have shown that the *EGFR* p.s768i mutation is sensitive to EGFR‐TKIs.^13^, ^14^, ^15^ A retrospective study suggested that among 6698 patients with adenocarcinoma, 35 patients had s768i mutations and 22 had combined sensitive mutations; the objective response rate (ORR) of lung cancer patients with p.s768i + p.l858r after EGFR‐TKI treatment was 60%.^16^ However, for S768I‐only mutants, the objective response rate was 40.0%.^16^ These results showed that the s768i mutation was sensitive to first‐generation TKIs, but the overall remission rate was still lower than that of the sensitive mutation. Oztan *et al*. identified the EGFR G724S variant in two T790M‐positive NSCLC patients whose disease progressed while on osimertinib treatment, suggesting that G724S is another potential acquired mutation leading to resistance to third‐generation EGFR‐TKIs. However, Peled *et al*. detected EGFR G724S in an NSCLC patient just after gefitinib treatment, and the G724S clone significantly increased during osimertinib treatment.^17^ The conclusions on the relationship between EGFR G724S and EGFR‐TKI are inconsistent.

*EGFR* s768i point mutation occurs in 1%–2% of *EGFR* mutant lung cancers, and can occur alone but often in combination with other *EGFR* sensitive mutations. *EGFR* G724S is an uncommon mutation that arises after treatment with first EGFR‐TKIs in NSCLC patients, as shown by the very low mutation frequency of 0.43% (five out of 1170) revealed in osimertinib treatment‐naive patients.^18^ Interestingly, these two rare mutations were found together in the same patient at the time of diagnosis. We consider that molecular‐targeted drugs are lacking in evidence of effectiveness, and the performance status is good (ECOG = 1). Therefore, we chose chemotherapy as the first‐line treatment, and the symptoms regressed and the tumors shrank as expected. However, the patient could not tolerate long‐term chemotherapy and refused further chemotherapy. The PFS of second‐line therapy (icotinib) was only one month, which may be related to the *EGFR* G724S mutation. The mechanisms are currently unclear. Previous studies have shown that afatinib might be the optimal EGFR‐TKI against these uncommon *EGFR* mutations.^15^ The efficacy of afatinib in the treatment of *EGFR* nonresistant rare mutations is not significantly different from that of sensitive mutations, which is better than that of the first generation of EGFR‐TKIs. Therefore, afatinib was selected for the third‐line treatment, and the PFS was six months. Subsequent results from genetic detection revealed the *EGFR* T790M mutation, and the EGFR G724S was still present. Approximately 50% of acquired drug resistance is related to the T790M mutation, and osimertinib is mainly aimed at patients with secondary T790M resistance mutations after TKI treatment. We chose osimertinib for the next treatment, but at the same time, we were concerned about the existence of EGFR G724s affecting therapeutic effects. Indeed, the patient developed symptoms of cough after 15 days, and took a turn for the worse. The patient refused genetic sequencing, and we were running out of treatment options. Peled *et al*. reported that in a white nonsmoking male with EGFR 19 exon deletion, EGFR 19 exon deletion, T790M and G724S appeared after disease progression. The T790M clone disappeared after treatment with osimertinib, and the number of g724s clones decreased significantly after treatment with a combination of afatinib and osimertinib.^17^ To target the *EGFR* G724S mutation, afatinib (30 mg daily) was added to osimertinib (80 mg daily). The result was not very satisfactory, and the disease progressed again after two months. We subsequently adjusted the dosage of afatinib because the patient was unable to tolerate the adverse reactions, and if administration could have continued without reducing the dose by controlling the side effects, a better result may have been achieved. The patient was then given treatment with anlotinib (12 mg, orally once daily for 14 days every three weeks). We will continue our follow‐up and look forward to a longer survival.

Because the number of patients with rare mutations is small and they have high heterogeneity, the efficacy of EGFR‐TKIs in patients with rare *EGFR* mutations remains unclear. However, a large number of clinical studies have shown that the efficacy of EGFR‐TKIs in patients with rare *EGFR* mutations is different, and the difference is great. This suggests that we should analyze these patients separately in clinical research and provide them with more effective individualized treatment. Afatinib might be the optimal EGFR‐TKI against these uncommon *EGFR* mutations. The urgent task is to develop and use a gene detection method that can recognize rare mutations of different *EGFRs* as soon as possible and, based on large‐scale prospective clinical research, to develop a reasonable and effective treatment plan for patients with rare *EGFR* mutations in order to achieve a better prognosis.
